# Advances in the synthesis and applications of macrocyclic polyamines

**DOI:** 10.1098/rsos.231979

**Published:** 2024-06-19

**Authors:** Yongguang Gao, Lina Guo, Xinhua Liu, Na Chen, Xiaochun Yang, Qing Zhang

**Affiliations:** ^1^ Department of Chemistry, Tangshan Normal University, Tangshan 063000, People’s Republic of China; ^2^ Hebei Key Laboratory of Degradable Polymers, Tangshan Normal University, Tangshan 063000, People’s Republic of China; ^3^ Tangshan Silicone Key Laboratory, Tangshan Normal University, Tangshan 063000, People’s Republic of China; ^4^ Tangshan First Vocational Secondary Specialized School, Tangshan 063000, People’s Republic of China

**Keywords:** macrocyclic polyamine, supramolecular chemistry, gene vector, fluorescent probe

## Abstract

Macrocyclic polyamines constitute a significant class of macrocyclic compounds that play a pivotal role in the realm of supramolecular chemistry. They find extensive applications across diverse domains including industrial and agricultural production, clinical diagnostics, environmental protection and other multidisciplinary fields. Macrocyclic polyamines possess a distinctive cavity structure with varying sizes, depths, electron-richness degrees and flexibilities. This unique feature enables them to form specific supramolecular structures through complexation with diverse objects, thereby attracting considerable attention from chemists, biologists and materials scientists alike. However, there is currently a lack of comprehensive summaries on the synthesis methods for macrocyclic polyamines. In this review article, we provide an in-depth introduction to the synthesis of macrocyclic polyamines while analysing their respective advantages and disadvantages. Furthermore, we also present an overview of the recent 5-year advancements in using macrocyclic polyamines as non-viral gene vectors, fluorescent probes, diagnostic and therapeutic reagents as well as catalysts. Looking ahead to future research directions on the synthesis and application of macrocyclic polyamines across various fields will hopefully inspire new ideas for their synthesis and use.

## 1. Introduction

Supramolecular chemistry has emerged as a burgeoning interdisciplinary field, integrating various disciplines including material science, pharmaceutical science, life science and modern chemistry [1–4]. Within the realm of supramolecular chemistry, macrocyclic compounds have gained significant attention and are currently one of the most actively researched areas in both chemistry and biomedicine [5–7]. Since the 1960s, extensive studies on macrocyclic compounds such as crown ethers, cyclodextrins, calixarenes, macrocyclic lactones, porphyrins, cyclic peptides, cucurbiturils, rotaxanes phthalocyanines and macrocyclic polyamines have propelled supramolecular chemistry to new heights. Macrocyclic polyamines are cyclic compounds composed of multiple amino groups. As an excellent ligand with a unique cavity structure, macrocyclic polyamines have found extensive applications such as chemical nucleases, biosensors, magnetic resonance imaging agents, fluorescent probes, DNA recognitions and enzyme simulation catalysts, immunotherapy drugs, and so forth [8–10]. Macrocyclic polyamines are cyclic alkane compounds containing at least three or more nitrogen atoms and nine or more total atoms. Among these polyamines, those with three or four nitrogen atoms are the most commonly encountered. Different publications may use varying nomenclature for macrocyclic polyamines For example, entry 1 in table 1 is referred to as 1,4,7-triazacyclononane in some publications while others use TACN or [9]aneN_3_. Following naming conventions, we present a summary of each macrocyclic polyamine’s name along with its corresponding CAS number and molecular formula in table 1 alongside relevant literature on synthesis or application. We believe that this compilation will be beneficial to readers engaged in related research.

**Table 1.** Information related to macrocyclic polyamines.

**e**ntry	**s**tructure	**n**ame	**a**bbreviation	CAS **n**o.	**f**ormula	**r**eference
1	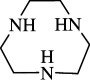	1,4,7-triaza cyclononane	TACN ([9]aneN_3_)	4730-54-5	C_6_H_15_N_3_	[11–22]
2	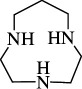	1,4,7-triaza cyclodecane	1,4,7-TACD ([10]aneN_3_)	56575-49-6	C_7_H_17_N_3_	[23]
3	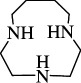	1,4,7-triaza cycloundecane	1,4,7-TACUD ([11]aneN_3_)	60563-32-8	C_8_H_19_N_3_	[24]
4	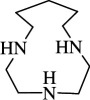	1,4,7-triaza cyclododecane	1,4,7-TACDD ([12]aneN_3_)	23635-83-8	C_9_H_21_N_3_	[24]
5	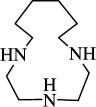	1,4,7-triaza cyclotridecane	1,4,7-TACTD ([13]aneN_3_)	52877-35-7	C_10_H_23_N_3_	[24]
6	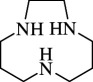	1,4,8-triaza cycloundecane	1,4,8-TACUD ([11]aneN_3_)	36532-31-7	C_8_H_19_N_3_	[24,25]
7	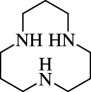	1,5,9-triaza cyclododecane	1,5,9-TACDD ([12]aneN_3_)	294-80-4	C_9_H_21_N_3_	[17,24–34,35–44]
8	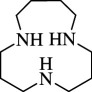	1,5,9-triaza cyclotridecane	1,5,9-TACTD ([13]aneN_3_)	54365-83-2	C_10_H_23_N_3_	[24]
9	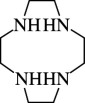	1,4,7,10-tetraaza cyclododecane	1,4,7,10-TACDD (cyclen, [12]aneN_4_)	294-90-6	C_8_H_20_N_4_	[5,45–47,23,26,48–65]
10	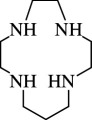	1,4,7,10-tetraaza cyclotridecane	1,4,7,10-TACTD ([13]aneN_4_)	295-14-7	C_9_H_22_N_4_	[48]
11	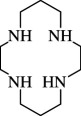	1,4,8,11-tetraaza cyclotetradecane	1,4,8,11-TACTD (cyclam or ([14]aneN_4_)	295-37-4	C_10_H_24_N_4_	[12,47,48,62,66–68,69–71]
12	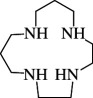	1,4,7,11-tetraaza cyclotetradecane	1,4,7,11-TACTD ([14]aneN_4_)	52877-36-8	C_10_H_24_N_4_	[68]
13	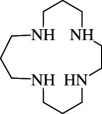	1,4,8,12-tetraaza cyclopentadecane	1,4,8,12-TACPD (Cyclal, [15]aneN_4_)	15439-16-4	C_11_H_26_N_4_	[26]
14	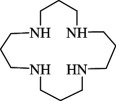	1,5,9,13-tetraaza cyclohexadecane	1,5,9,13-TACHD ([16]aneN_4_)	24772-41-6	C_12_H_28_N_4_	[68]
15	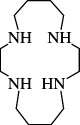	1,4,9,12-tetraaza cyclohexadecane	1,4,9,12-TACHD ([16]aneN_4_)	70072-64-9	C_12_H_28_N_4_	[72]
16	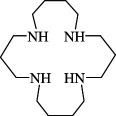	1,5,10,14-tetraaza cyclooctadecane	1,5,10,14- TACOD ([18]aneN_4_)	68966-28-9	C_14_H_32_N_4_	[72]
17	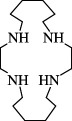	1,4,11,14-tetraaza cycloicosane	1,4,11,14-TACI ([20]aneN_4_)	296-68-4	C_16_H_36_N_4_	[72]
18	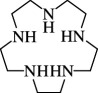	1,4,7,10,13-pentaazacyclo pentadecane	1,4,7,10,13-PACPD ([15]aneN_5_)	295-64-7	C_10_H_25_N_5_	[23,61]
19	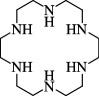	1,4,7,10,13,16-hexaazacyclo octadecane	1,4,7,10,13,16-HACOD ([18]aneN_6_)	296-35-5	C_12_H_30_N_6_	[23]
20	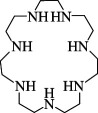	1,4,7,10,13,16,19-heptaazacyclo henicosane	1,4,7,10,13,16,19-HACH ([21]aneN_7_)	296-85-5	C_14_H_35_N_7_	[23]

In macrocyclic polyamines, the unbonded lone electrons of the nitrogen atoms forming the ring can not only form ammonium ions with hydrogen ions but also interact with metal ions to create stable metal complexes. Moreover, macrocyclic polyamines can form supramolecular compounds through various non-covalent bond interactions such as hydrogen bonding, van der Waals forces, hydrophobic interactions and π–π stacking interactions with positively charged ions or negatively charged organic anions as host and guest molecules. This enables specific recognition of organic small molecules and biological molecules including amino acids, proteins and nucleic acids. Compared with linear polyamine compounds, macrocyclic polyamines exhibit stronger pre-assembly ability and recognition capacity for guest molecules owing to variations in ring cavity size as well as the number and type of electron donor atoms within the ring structure. Consequently, macrocyclic polyamines have found extensive applications in chemical nucleases, biosensors, biomimetic enzyme catalysts and photodynamic therapy and are gradually emerging as a key research area in macrocyclic chemistry [45,73,74]. This review article primarily focuses on synthesizing methods for macrocyclic polyamines containing 3–7 nitrogen atoms while discussing their advantages and disadvantages in detail. Additionally, the advancements of the last 5 years in using these macrocycles as non-viral gene vectors, fluorescent probes, diagnostic agents, therapeutic reagents and catalysts are discussed aiming to provide insights into the design, synthesis and application of novel macrocyclic polyamines.

## 2. Synthesis of macrocyclic polyamines

### 2.1. Cycloaddition of dihalides and amines

#### 2.1.1. Cycloaddition of dihalides and unprotected diamines

**Scheme 1.**
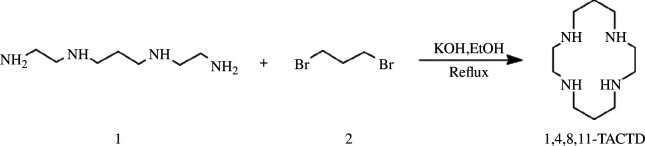
Synthesis of 1,4,8,11-TACTD.

1,4,8,11-Tetraazacyclotetradecane (1,4,8,11-TACTD), commonly known as cyclam or [14]aneN_4_ in literature, is a macrocyclic polyamine that has been extensively investigated in early studies. Its synthesis was initially reported by Alphen through the reaction between 1,3-dibromopropane **2** and 1,3-bis(2'-aminoethylamino)-propane **1** in the presence of caustic potash (scheme 1; [66]). However, owing to the nascent stage of research on macrocyclic chemistry, the synthesis of cyclam did not receive significant attention. It was only after three chemists, Pedersen, Lehn and Cram, were awarded the Nobel Prize in chemistry for their contributions to crown ether-related supramolecular chemistry in 1987 that the synthesis of macrocyclic polyamines gained prominence.

**Scheme 2.**
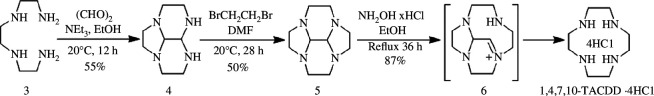
Synthesis of 1,4,8,11-TACDD hydrochloride.

The synthesis of macrocyclic polyamines through the aforementioned method necessitates a prolonged reaction time in an ultra-dilute solution. Sandnes *et al*. [75] devised a straightforward three-step synthesis of (1,4,7,10-tetraazacyclododecane) TACDD from triethylenetetraamine and glyoxal (scheme 2). The bis-aminal **4** is obtained by reacting a 40% aqueous glyoxal solution with triethylenetetramine in ethanol. Ring-forming dialkylation using 1,2-dibromoethane yields tetraamine **5** with a yield of 50%. Hydrolysis of compound **5** with excess hydroxylamine hydrochloride furnishes the tetrahydrochloride salt of 1,4,7,10-TACDD. The crude products are subsequently recrystallized in cold ethanol to achieve an overall yield of 87%. However, this synthetic approach for macrocyclic polyamine formation yields low amounts of the desired product and exhibits only a total yield of merely 24% over the three-step process, the resulting salt formed between four molecules of hydrogen chloride and 1,4,7,10-TACDD demonstrates excellent stability against oxidation by air and can be stored for extended periods. Argese *et al*. [46] discovered that various isomers of bis-aminals possessing distinct structures could be generated via the reaction between triethylenetetramine **3** and glyoxal. However, the subsequent steps involving amination, reduction and hydrolysis reactions still fail to provide satisfactory yields.

**Scheme 3.**
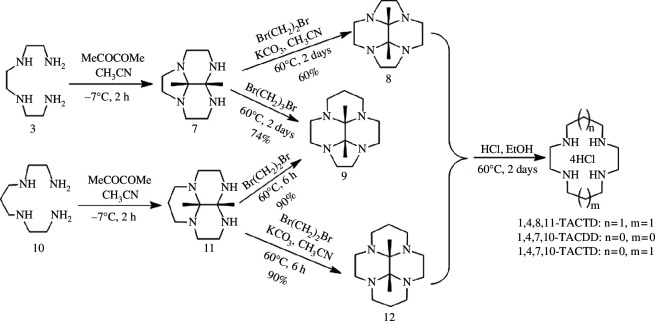
Synthesis of 1,4,8,11-TACTD, 1,4,7,10-TACDD and 1,4,7,10-TACTD hydrochloride.

Hervé *et al*. [47] synthesized three macrocyclic polyamines, namely 1,4,8,11-TACTD, 1,4,7,10-TACDD and 1,4,7,10-TACTD (scheme 3), by reacting with triethylenetetramine **3** or *N*,*N*'-bis(2-aminoethyl)-1,3-propanediamine **10** using butanedione instead of glyoxal. Butanedione reacts with compound **3** and compound **10** in CH_3_CN to give the tricyclic bis-aminals **7** and **11**, respectively. The reaction of a dibromo-derivative (1,2-dibromoethane or 1,3-dibromopropane) with **7** and **11** in CH_3_CN gives the protected macrocycles **8**, **9** and **12** in good yields. Notably, the butanedione-protected macrocycle **9** can be obtained either from **7** or **11** with retention of the *cis*-configuration. After improvement, the yield of bis-amines **7** and **11** in the cycloaddition reaction with dibromide is higher, reaching up to 90%. Tetraamines **8**, **9** and **12** can be hydrolysed into products in an acidic ethanol solution. Compared with Sandnes’s method, the by-products are reduced and the total yield is improved.

**Scheme 4.**
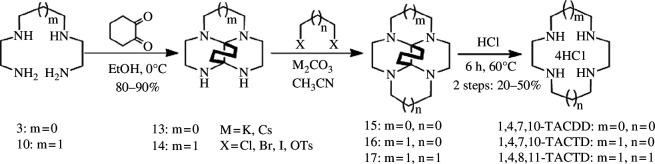
Synthesis of 1,4,8,11-TACTD, 1,4,7,10-TACDD and 1,4,7,10-TACTD hydrochloride by Prokov.

Recently, Prokhorov *et al*. have discovered a novel and facile synthetic route for the preparation of 1,4,8,11-TACTD, 1,4,7,10-TACDD and 1,4,7,10-TACTD (scheme 4; [48]). The condensation of the appropriate linear tetraamine with cyclohexanedione followed by cyclization of the resulting bisaminal intermediates (**13** and **14**) using dibromo- or ditosyloxy-derivatives afforded compounds **15**, **16** and **17**. Notably, using caesium carbonate as a proton trapper instead of potassium carbonate significantly enhances the yields in the cyclization step. Compared with previous methods based on bisaminal chemistry for synthesizing these macrocyclic polyamines (1,4,8,11-TACTD, 1,4,7,10-TACDD and 1,4,7,10-TACTD), this new approach using cyclohexanedione and caesium carbonate represents a substantial improvement: starting from linear tetraamine precursors and undergoing three protection–cyclization–deprotection steps results in desired products with yields ranging from 75% to 80%. Furthermore, the removal and recovery of protective groups can be achieved cleanly and quantitatively under mild acidic conditions.

**Scheme 5.**
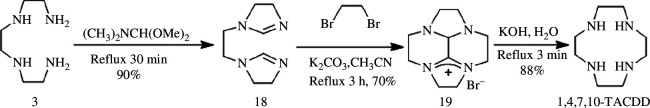
Synthesis of 1,4,7,10-TACDD by Athey Scheme 6 [49].

*N*,*N*-dimethylformamide dimethyl acetal (DMF-DMA) is a crucial intermediate in the synthesis of heterocyclic compounds, capable of efficiently converting primary amines and primary amides into amidine and acylamidine, respectively (Scheme 5) [76]. It finds wide-ranging applications in the synthesis of indole, 1,2,3-triketones and quinolones. Athey & Kiefer [49] used DMF-DMA to convert triethylenetetramine into bis-imidazoline **18** with over 90% yield. In the presence of potassium carbonate, compound **18** undergoes a cyclization reaction with dibromoethane to obtain monoimidazole compound **19** at a yield of 70%. Excessive cyclization time can lead to symmetric diacritical oximidinium production. Compound **19** can be refluxed in potassium hydroxide solution for half an hour to obtain high-yield (88%) 1,4,7,10-TACDD. Compared with previous methods for synthesis, Athey’s method has mild reaction conditions and high yields that enable large-scale production.

**Scheme 6.**
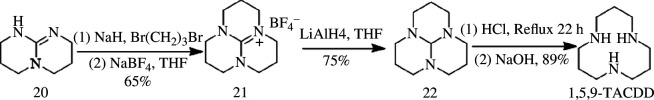
Synthesis of 1,5,9-TACDD by Alder [77].

In addition to the macrocyclic polyamine with four nitrogen atoms, 1,5,9-TACDD containing three nitrogen atoms can also be obtained by [1+1] ring addition of unprotected diamines and dihalides. Alder *et al*. [77] used bicyclic guanidine 1,5,7-triazabicyclo[4.4.0]dec-sene **20** as a template for cyclization with 1,3-dibromopropane in the presence of NaH followed by reaction with NaBF_4_ to yield tetrafluoroborate **21** in a yield of 65% (Scheme 6). Tetrafluoroborate **21** can be subsequently reduced to orthoamine **22** in tetrahydrofuran (THF) and refluxed for 22 h in the presence of 3 M HCl to afford a synthesis yield of 89% for obtaining 1,5,9-TACDD. Modifications of this procedure enable the preparation of other tricyclic guanidinium. This synthetic route provides an appealing alternative for synthesizing macrocyclic triamines and may have broader applications in constructing macrocycles around covalently bound template atoms or groups. However, it should be noted that this method requires stringent conditions including anhydrous and oxygen-free environments while handling challenging reagents such as NaH and LiAlH_4_.

#### 2.1.2. Cycloaddition of dihalides and protected diamines

**Scheme 7.**
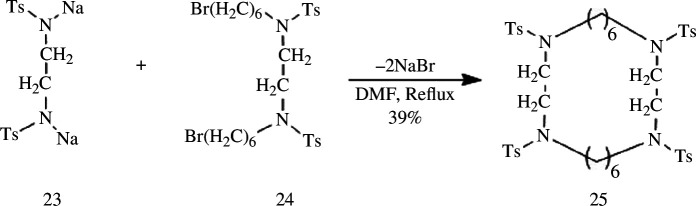
Synthesis of macrocyclic polyamine **25** by Stetter.

In general, the exposed amino group is highly susceptible to oxidation reactions in ambient air, rendering, a common synthetic approach for macrocyclic polyamines involves initial protection of the amino group followed by cycloaddition reactions. Commonly used amino protection groups include Ts-, Ms-, Boc-, Bn- and Ac-. Stetter *et al*. [78] discovered that Ts-protected diphenyl-*o*-diamine can undergo cyclization reaction with dibrominates under alkaline conditions (Scheme 7). In the subsequent year, they replicated this methodology and synthesized the first Ts-protected macrocyclic polyamine **25** containing four nitrogen atoms through a one-step ring-closing reaction using Ts-protected ethylenediamine disodium salt **23** and dibromide **24** as starting materials [79].

**Scheme 8.**
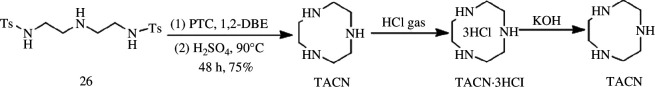
Synthesis of TACN by Kanwar.

Kanwar *et al*. [11] enhanced the synthesis methodology of Stetter by using Ts-protected diethylenetriamine **26** in the presence of phenylthiocarbamide (PTC) and potassium carbonate to undergo a cycloaddition reaction with 1,2-dibromoethane (1,2-DBE) (Scheme 8), followed by heating under concentrated sulfuric acid conditions to obtain an air-sensitive liquid TACN characterized by a pungent odour. Introduction of dry hydrogen chloride gas yields stable hydrochloride. Prior to usage, removal of hydrogen chloride using potassium hydroxide allows for isolation of free TACN.

#### 2.1.3. Cycloaddition of dihalides and monoamines

**Scheme 9.**
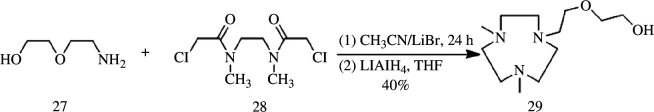
Synthesis of *N*-alkylated TACN. OTs, Tosyl group (p-CH3-C6H4-SO2-); OMs, methylsulfonyl group (CH3-SO2-).

The cycloaddition of diaminodihalides with monoamines is a commonly used synthetic method for the production of *N*-alkylated macrocyclic polyamines. Owing to the modification of all nitrogen atoms by alkyl groups, macrocyclic polyamines exhibit enhanced stability against oxidation in ambient air and can be stored at low temperatures for extended periods. Chloroacetyl chloride serves as a crucial reagent in the synthesis of amidated dichlorides, which undergo amination reactions with *N*-alkylated diamines to yield dichloro-substituted amides. Subsequently, these amides engage in cycloaddition reactions with monoamine derivatives. Reduction of the resulting amide products using lithium aluminium hydride or borane leads to the formation of *N*-alkylated macrocyclic polyamines. Bis(2-chloro-*N*-methylacetamide) **28** was synthesized by Bradshaw *et al*. [12] using chloracetyl chloride and *N*,*N*'-dimethylethylenediamine as starting materials (Scheme 9). In the presence of sodium carbonate and lithium bromide, refluxing 2-(2-aminoethoxy)ethanol **27** in acetonitrile for 24 h followed by reduction with lithium aluminium tetrahydride afforded *N*-[2-(2-hydroxyethoxy)ethyl]-substituted TACN **29**. In the cyclization reaction, isopropyl alcohol is required for column chromatography purification, and the purification effect is not ideal. Isopropyl alcohol was required during column chromatography purification in the cyclization reaction. However, its purification efficacy was suboptimal.

Subsequently, Krakowiak *et al*. [13] expanded the reaction substrates bis(2-chloro-*N*-methylacetamide) and monoamines to synthesize a series of tertiary (aminoalkyl)-substituted TACN and 1,4,7,10,13-pentaazacyclopentadecane derivatives. In an attempt to enhance the yield, they substituted isopropyl alcohol with a mixture of methanol and ammonia for crude product purification but obtained unsatisfactory results. Furthermore, they explored reversed-phase ion exchange chromatography as a purification method; although it improved the yield significantly, its high cost limited its practicality. More recently, this methodology has been used for synthesizing polymers containing 1,4,7-triazacyclononane (TACN) as well as chiral ligands and phosphate ester cleavage products; however, the yields achieved remain suboptimal [14–16]. The key advantage of this approach lies in obviating the need for ultra-dilute solutions during cycloaddition reaction.

### 2.2. Cycloaddition of disulfonates and amines

#### 2.2.1. Disulfonates and Ts-protected diamines

In addition to halogens, sulfonic acid esters serve as excellent leaving groups, with common examples including methyl sulfonate and *p*-toluenesulfonate. The cycloaddition reaction of disulfonates with protected diamines represents the prevailing approach for synthesizing macrocyclic polyamines. This versatile method enables the synthesis of macrocyclic polyamine derivatives containing 3–7 nitrogen atoms. To enhance the nucleophilicity of nitrogen atoms, bissulfonamide sodium salts are typically formed by deprotonating the diamine.

**Scheme 10.**
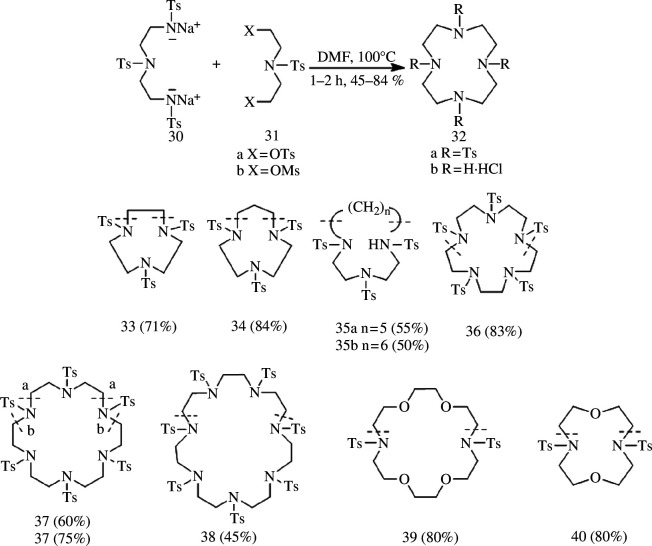
Synthesis of Ts-protected macrocyclic polyamine by Richman and Atkins.

Richman & Atkins [23] used this methodology to react the tritosyl derivative of diethylenetriamine with disulfonic acid esters in DMF at 100°C for 1–2 h. A range of macrocyclic polyamines encompassing diverse nitrogen atoms has been successfully synthesized using this approach (Scheme 10), which is also applicable for constructing macrocyclic heterocyclic compounds containing both nitrogen and oxygen atoms. Throughout the reaction, all nitrogen atoms are protected by *p*-toluenesulfonyl chloride; subsequent removal of the protective group is achieved *via* concentrated sulfuric acid treatment followed by treatment with 6 M hydrochloric acid to obtain the corresponding macrocyclic polyamine hydrochloride, yielding between 45% and 84%. Briellmann *et al*. [24] reported the synthesis of a series of macrocycles ranging from 9 to 13 members using the *p*-toluenesulfonate method; however, it was observed that numerous unidentified by-products were generated during the cyclization process with yields ranging from 23% to 57%. Regrettably, these by-products have not been isolated or characterized thus far. The sulfonate-based synthesis of macrocyclic polyamines obviates the need for ultra-dilute without requiring template induction, thereby demonstrating its broad applicability in synthesizing diverse heteroatom-containing macrocyclic polyamines.

In the method proposed by Richman and Atkins, the crude product resulting from the reaction between *p*-tolylsulfonamide and sodium ethanol is subjected to recrystallization in ethanol for obtaining bissulfonamide sodium. In the two-phase system of inorganic bases (LiOH, NaOH or KOH) and toluene, Lukyanenko *et al*. [26] discovered that acyclic bis(sulphonamide)s and dibromides or ethylene glycol bis(toluene-*p*-sulphonate)s can undergo direct ring closure with the presence of phase transfer catalysts, resulting in the formation of macrocyclic polyamines containing 2, 3 and 4 nitrogen. The catalytic activity of phase transfer catalysts follows the order: Bu_4_NI ≈ Bu_4_NBr > Bu_4_NCl > Bu_4_NHSO_4_ > Et_3_NCH_2_C_6_H_5_NCl. Tetrabutylammonium iodide and tetrabutylammonium bromide exhibit high catalytic activity, which can be attributed to their excellent thermal stability in alkaline media. Bell *et al*. [27] synthesized a series of [12]aneN_3_ derivatives using NaH instead of NaOH in DMF. Owing to its good solubility in DMF, NaH can react without the need for a phase transfer catalyst, resulting in an average yield of approximately 30%. Inorganic bases such as potassium carbonate and caesium carbonate also significantly influence the cyclization yield [16,80–82]. In general, the weaker the alkalinity, the longer the reaction time required and the reaction generally needs to be carried out in a dilute solution.

**Scheme 11.**
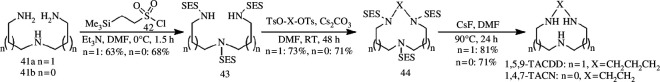
Synthesis of 1,5,9-TACDD and 1,4,7-TACN SES (trimethylsilylethanesulfonamides).

Except for methanesulfonyl chloride and *p*-toluenesulfonyl chloride, *β*-trimethylsilylethane-sulfonyl chloride **42** is occasionally used as an amino group protecting agent in cyclization reactions. Hoye *et al*. [17] discovered that *β-*trimethylsilylethanesulfonamides (SES) can undergo [1+1] cyclization reaction with disulfonate in the presence of caesium carbonate (Scheme 11). In comparison to methylsulfonyl chloride and *p*-toluenesulfonyl chloride, SES exhibits milder deprotection conditions. Macrocyclic polyamines protected by SES can be deprotected in the presence of CsF. Furthermore, macrocyclic polyamines synthesized by this method eliminate the need for ultra-dilute solutions, resulting in yield of approximately 50% for the two-step cyclization and deprotection process.

#### 2.2.2. Disulfonates and unprotected diamines

**Scheme 12.**
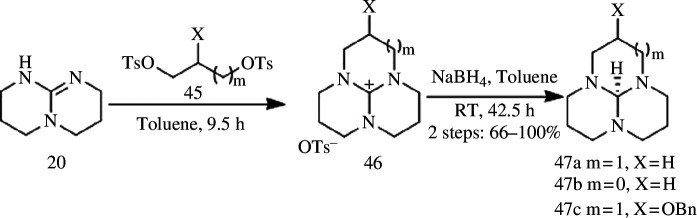
Synthesis of macrocyclic triamines **47a–47c**.

Owing to the inherent instability and susceptibility to oxidation by air, direct cyclization of unprotected diamines with disulfonates is rarely observed. Kim *et al*. [25] successfully synthesized tricyclic orthoamides **46** through a one-pot method using bicyclic guanidine **20** as the starting material (Scheme 12), followed by refluxing for 9.5 h under concentrated sulfuric acid conditions. This approach yields macrocyclic triamines **47a**–**47c**. It should be noted that the synthesis of macrocyclic triamine using this method is limited to the use of *p*-toluene sulfonate as the leaving group. Furthermore, sulfonates can only be attached to primary carbon atoms, and when attached to secondary carbon, neopentyl carbon, allyl carbon or benzyl carbon, cycloaddition reactions cannot be facilitated by means of this methodology.

### 2.3. Cycloaddition of diacids and unprotected diamines

**Scheme 13.**
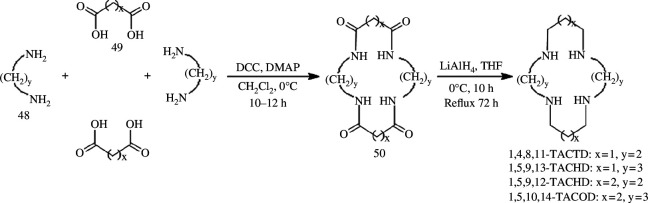
Synthesis of 1,4,8,11-TACTD, 1,5,9,13-TACHD (tetraazacyclohexadecane), 1,5,9,12-TACHD and 1,5,10,14-TACOD (tetraazacyclooctadecane).

The condensation reaction between carboxylic acids and amines represents a widely used approach for the synthesis of amides, which can be subsequently reduced to amine compounds using lithium aluminium hydride or borane as reducing agents. Chaudhary & Singh [50] used 4-dimethylaminopyridine (DMAP) as a catalyst along with 1,3-dicyclohexylcarbodiimide (DCC) as a condensing agent to react diamine with malonic or succinic acid in dichloromethane for 10–12 h (Scheme 13). This resulted in the formation of a series of cyclic tetramides, which were further subjected to reduction by lithium aluminium hydride, leading to the generation of corresponding macrocyclic polyamines.

They synthesized a series of tetraamides containing 14–18 atoms using the aforementioned method and used them for investigating the antibacterial and anti-inflammatory activities in conjunction with manganese ion complexes [72]. Denat *et al*. [83] subsequently enhanced this methodology by using oxalic acid instead of diamine to react with tetramine, resulting in the synthesis of macroyclic polyamines comprising eight nitrogen atoms. The improved approach exhibits simplicity in post-processing, obviates the need for column chromatography purification and eliminates protection or deprotection steps, rendering it suitable for synthesizing macrocyclic polyamines containing four or eight nitrogen atoms.

### 2.4. Cyclization of other reagents and diamines

#### 2.4.1. Malonate derivatives and diamines

**Scheme 14.**
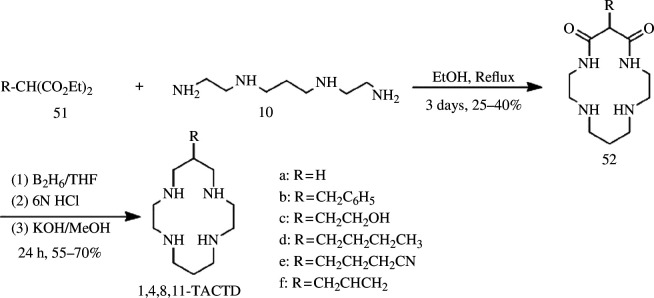
Synthesis of C3-substituted 1,4,8,11-TACTD derivatives.

Diethyl malonate serves as a crucial intermediate in organic synthesis owing to its facile formation of carbanions and susceptibility to acylation, alkylation and Michael reactions. Additionally, it can undergo exchange reactions with lower-grade amines to yield amides. Tabushi *et al*. demonstrated the direct cyclization of unprotected diamines with malonate derivatives instead of dibromides or disulfonic acid esters (Scheme 14), resulting in a series of *C*3-substituted 1,4,8,11-TACTD derivatives that were obtained via refluxing under borane conditions for 24 h [67]. Subsequently, Grisenti *et al.* [68] used this method to synthesize 1,4,8,12-tetraazacyclopentadecane (TACPD) and 1,5,9-13-tetraazacyclohexadecane (TACHD). However, the yield was only 10%.

#### 2.4.2. Oxamide/dithiooxamide and diamines

**Scheme 15.**
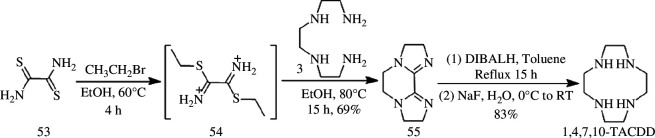
Synthesis of 1,4,7,10-TACDD by Weisman.

The bis-imidazoline compounds can be synthesized by cycloaddition of oxamide or dithioxamide with triethylenetetramine **3**, followed by reduction using lithium aluminium tetrahydride or lithium diisopropyl hydride to obtain macrocyclic polyamines [84]. Weisman & Reed [51] developed an efficient two-step synthesis of 1,4,7,10-TACDD from triethylenetetramine **3** and dithiooxamide **53** (Scheme 15). 1,4,7,10-TACDD precursor **55** was prepared in a yield of 69% through *S-*alkylation of dithiooxamide **53** with excess bromoethane, followed by reaction between the putative bis-thioimido ester salt **54** and triethylenetetramine **3**. This new synthesis provides a two-step route to obtain 1,4,7,10-TACDD with an overall yield of 57%. No protecting groups are necessary and the solvent requirements are relatively modest compared with the Richman–Atkins synthesis.

#### 2.4.3. Oxaldehyde/chloroacetaldehyde and diamines

**Scheme 16.**
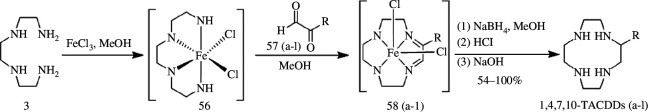
Synthesis of C2-substituted 1,4,7,10-TACDD derivatives. R=^t^Bu (a);Ph (b);4-NO_2_-Ph (c);4-MeO-Ph (d);4-Cl-Ph (e);4-Br-Ph (f);4-CF3-Ph (g) thiophen-2-yl (h);naphthalen-2-yl (i);3,5-bis(trifluoromethyl)phenyl (j);2-fluorenyl (k);phenanthren-2-yl(l).

Triethylenetetramine **3** possesses four nitrogen atoms and exhibits the ability to coordinate with metal ions. Reid *et al*. [52] used Fe^3+^-triethylenetetraamine complexes **56** as a template for reacting with glyoxal containing diverse substituents **57 a**–**57l** to synthesize a series of *C*2-substituted-1,4,7,10-TACDDs (Scheme 16). In addition, macrocyclic polyamines containing amide bonds were obtained by B(III) or Sb(III)-templated intramolecular ammonolysis reaction of triamino or tetraamino esters [18,85,86]. These lactamated macrocyclic polyamines play crucial roles as intermediates in the synthesis of macrocyclic spermidine and spermine alkaloids such as (*S*)-(±)-dihydroperiphylline, (±)-buchnerine, (±)-verbacine, (±)-verbascenine and (±)-verbaskine [87].

**Scheme 17.**
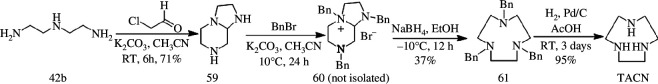
Synthesis of TACN.

The reaction between diethylenetriamine and chloroacetaldehyde affords the bicyclic aminal intermediate in high yield, which not only reacts with halogenated hydrocarbons to obtain *N*-functionalized TACN derivatives but also reacts with sodium cyanide to form *C*-functionalized TACN derivatives (Scheme 17; [88]). Subsequent reduction of the bicyclic aminal with sodium borohydride, followed by removal of the benzyl protecting group, yields unprotected TACN with an overall reaction yield of approximately 25%. However, this method is limited to the synthesis of single-atom functionalized TACN and proves challenging for polyatomic functionalized TACN and other macrocyclic polyamines.

## 3. Applications of macrocyclic polyamines

### 3.1. Gene vectors

Macrocyclic polyamines represent a crucial class of organic molecules that exhibit multiple modifiable sites, possess the ability to coordinate with diverse metals and offer the advantages of excellent water solubility. Consequently, they find extensive applications in gene vectors. Gene vectors encompass both viral and non-viral vectors, with viral vectors being predominantly used in gene therapy regimens (approx. 70% utilization rate). Various retroviruses, adenoviruses, herpesviruses and pox viruses have been used as viral vectors [28–30]. Notably, adenovirus and retroviral vectors have demonstrated some success in cancer therapy during early clinical trials. However, these viral vectors suffer from several drawbacks including adverse immune responses, limited scalability for mass production, low payload capacity, inadequate targeting capabilities along with high costs. Consequently, there is growing interest in developing more efficient and safer non-viral gene delivery vectors [31]. Non-viral vectors offer the advantages of minimal immune response, facile customization and suitability for large-scale production, thereby presenting extensive prospects for application.

**Figure 1.**
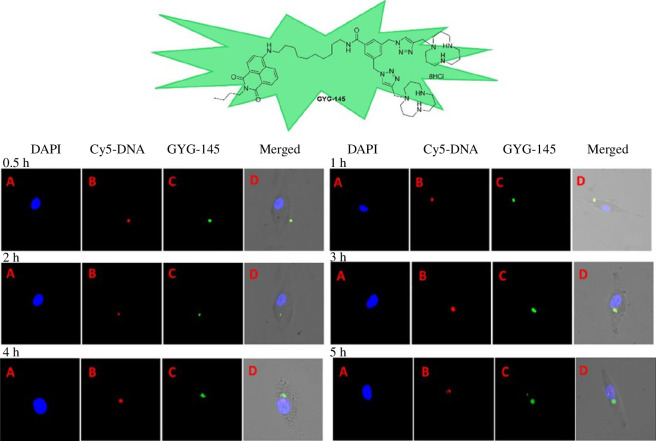
Real-time monitoring of DNA delivery in cells using a naphthimide-modified [12]aneN_3_ gene vector [34]. DAPI, 4',6-diamidino-2-phenylindole. GYG-145, naphthimide-modified [12]aneN3 gene vector. Copyright 2015 Royal Society of Chemistry.

1,5, 9-Triazacyclododecane, also known as 1,5,9-[12]aneN_3_, can interact with DNA through electrostatic or insertional mechanisms and is thus used for DNA delivery and biomimetic enzyme catalysis. Gao *et al*. [32] synthesized a series of alkyl chains-modified derivatives of 1,5,9-[12]aneN_3_ varying lengths that enable the condensation of DNA into spherical nanoparticles ranging from 130 to 220 nm. Among these derivatives, the one modified with unsaturated alkanes containing 18 carbon atoms exhibits superior transfection efficiency compared with commercial lipofectamine 2000. The incorporation of fluorescent naphthalimide enhances gene transfection and enables real-time tracking of DNA transport (figure 1; [33,34]). Furthermore, the conjugation of naphthalimide with cholic acid, a liver-targeting molecule, can effectively enhance DNA uptake in liver cancer cells while significantly reducing cytotoxicity [35]. Structure–activity relationship studies reveal that an increased distance between naphthalimide and [12]aneN_3_ leads to higher transfection efficiency [36]. Naphthalimide-modified [12]aneN_3_ derivatives not only serve as gene carriers for delivering DNA and RNA while monitoring their intracellular trafficking but also function as organelle dyes for lysosomes [37]. Additionally, they can be used as fluorescent probes for copper ion identification and ATP detection [38].

Lu *et al*. [39] primarily focus on the research of multifunctional non-viral gene vectors based on [12]aneN_3_. They synthesized triphenylamine-benzylideneimidazolone and hydrophobic alkyl chain-modified non-viral gene vectors, which exhibited remarkable two-photon properties, large stokes shifts and strong aggregation induction effects. These vectors not only efficiently discriminate single-stranded DNA and double-stranded DNA with varying lengths but also enable tracking of DNA transport in cells using two-photon fluorescence. Moreover, their transfection efficiency surpasses that of the commercial transfection reagent lipofectamine 2000. After benzothiazole modification, [12]aneN_3_ demonstrates excellent mitochondrial targeting ability with a 99% overlap rate compared with commercial mitochondrial staining reagents [40]. The transfection efficiency is 1.7 times higher than that of lipofectamine 2000 in the presence of cell-penetrating peptide. Recently, they synthesized a series of [12]aneN_3_-based non-viral vectors such as gemini-type, triphenylamine pyrazine and acenaphtho[1,2-b]quinoxaline with two-photon fluorescence characteristics. All these vectors exhibit superior fluorescence imaging properties along with exceptional serum stability and gene transfection ability, showcasing their immense potential for application in gene diagnosis and therapy [53–56].

The compound 1,4,7,10-tetraazacyclododecane (cyclen, 1,4,7,10-[12]aneN_4_) possesses four amino groups that exhibit resistance to foreign acids and bases and demonstrate a proton sponge effect. Consequently, [12]aneN_4_-based non-viral gene vectors have garnered significant attention. Yu *et al*. synthesized a series of cationic liposomes based on [12]aneN_4_ with imidazole groups which significantly enhance transfection activity in the presence of calcium ions [57–59]. When an oleyl group containing unsaturated bonds is used as the hydrophobic moiety, the transfection efficiency in the presence of calcium ions can reach up to five times that of lipofectamine 2000. Furthermore, they also developed cationic liposomes with excellent biocompatibility and degradable disulfide bonds that achieve comparable transfection efficiency to commercial lipofectamine 2000 [60].

Recently, it has been discovered that Zn^2+^-[12]aneN_4_ complexes modified with alkyl possess the ability to concentrate DNA into nanoparticles and safeguard it against degradation by nucleases [89,90]. In comparison to uncoordinated [12]aneN_4_ molecules, the Zn^2+^-[12]aneN_4_ complex exhibits significantly enhanced lysosomal escape capability, thereby augmenting gene transfection efficiency. The double alkyl chain-modified Zn^2+^-[12]aneN_4_ complex demonstrates superior delivery potential by facilitating the transportation of not only plasmid DNA but also small interfering (siRNA) and proteins [45]. Furthermore, the coordination between diglycidyl ether and [12]aneN_4_ with Zn^2+^ not only enhances transfection efficiency but also greatly improves resistance to serum [91]. These fluorine atom-modified Zn(II) polymers exhibit enhanced endocytosis and lysosomal escape abilities, rendering their DNA and protein delivery effects comparable to those of commercial 25 kDa polyethyleneimine [92].

### 3.2. Fluorescent probes

Fluorescent probes, as a new technological means that emerged in the 1980s, have been widely applied in various fields such as disease diagnosis, environmental monitoring and life sciences [93–96]. Compared with other traditional detection techniques such as atomic absorption spectroscopy, atomic emission spectroscopy and mass spectrometry, fluorescence probes have the advantages of high sensitivity, good selectivity, simple operation, *in situ* detection, minimal damage to organisms and visualization. As a new efficient and convenient detection method, fluorescent probes play a very important role in the detection of metal ions, phosphate anions and small biological molecules [97–100].

Fluorescent probes typically consist of three components: a fluorescence unit, a recognition unit and a connection unit. The primary role of the fluorescence unit is to generate a fluorescence signal, which undergoes changes in intensity or emission wavelength upon interaction with the guest molecule, thereby serving as an expression of the signal. Commonly used fluorescence units include naphthalimide, rhodamine B, coumarin and BODIPY [19,101–103]. Recognition units selectively identify guest molecules through coordination, hydrogen bonding and chemical reactions. Macrocyclic polyamines, DPA and crown ethers are frequently used recognition units. The connection unit facilitates effective transmission of the fluorescence signal by linking the fluorescence unit with the recognition unit.

#### 3.2.1. Metal ion probes

Zinc is a crucial trace element in the human body, playing a pivotal role in numerous vital biochemical processes [104]. It actively contributes to bodily growth and development, regulates normal appetite, boosts immune function and facilitates wound healing. Inadequate zinc levels can result in diminished appetite, impaired digestion and compromised immunity. Severe zinc deficiency may lead to stunted growth and enterogenic limb dermatitis. Henceforth, it holds immense significance to develop precise and rapid detection probes for zinc ions that aid in diagnosing and treating conditions associated with zinc insufficiency.

Garau *et al*. synthesized benzene ring and quinoline-modified [9]aneN_3_ (TACN) derivatives, investigating their metal ion recognition capabilities towards Cd^2+^, Co^2+^, Cu^2+^, Fe^3+^, Hg^2+^, K^+^, Mg^2+^, Mn^2+^, Ni^2+^, Zn^2+^ and Pb^2+^[105]. The quinoline-modified [9]aneN_3_ probe with urine as the connection unit exhibits high fluorescence enhancement effects on four metal ions Cd^2+^, Zn^2+^, Pb^2+^ and Cu^2+^. The [9]aneN_3_ probe modified with quinoline as the linker exhibits significant fluorescence enhancement effects specifically for Cd^2+^, Zn^2+^, Pb^2+^ and Cu^2+^. Moreover, the amide-linked [9]aneN_3_-based probe selectively responds to Zn^2+^ ions, displaying a remarkable increase in fluorescence intensity upon the addition of Zn^2+^. Mechanistic studies reveal that Zn^2+^form 1:1 and 1:2 coordination complexes with [9]aneN_3_, while the carbonyl group in the amide bond cannot coordinate with zinc ions. Owing to its high rigidity and limited water solubility, metal ion recognition experiments are conducted in a MeCN/H_2_O (4 : 1, v/v) mixed solution. Furthermore, Savastano *et al*. [20] successfully synthesized a water-soluble Zn^2+^ fluorescent probe, incorporating anthracene as the chromophore and [9]aneN_3_ as the recognition unit. This probe exhibits excellent selectivity, even in the presence of numerous interfering anions.

Iron is a vital trace element in the human body, with its concentration ranking highest among all trace elements. It is a constituent of haemoglobin and myoglobin, serving as a carrier for oxygen and carbon dioxide during the metabolic processes within the human body [106]. However, excessive intake of Fe^3+^ can lead to intracellular elastin damage, resulting in arterial wall calcification or hardening [107,108]. Therefore, the recognition of Fe^3+^ has remained a prominent research focus for scientists. Lin *et al*. [109] synthesized a green self-luminescent compound *N*-5-acetyl-2-hydroxy-benzamide-1,4,7-triazacyclononane (btacn), which exhibited high sensitivity towards metal ions. The fluorescence intensity of btacn underwent significant changes upon addition of Cu^2+^, Co^2+^, Zn^2+^, Mn^2+^ and Fe^3+^ ions. Notably, the interaction between (btacn) and ZnCl_2_ results in the formation of a selective complex, Zn(btacn)Cl_2_, with excellent affinity for Fe^3+^. Remarkably, the introduction of Fe^3+^ leads to a substantial enhancement in fluorescence intensity as well as a redshift of approximately 20 nm in Zn(btacn)Cl_2_. Mechanistic investigations suggest that electrostatic attraction may facilitate the interaction between Fe^3+^ and oxygen atoms within btacn. Most notably, Zn(btacn)Cl_2_ exhibits excellent selectivity towards Br^−^ ions and the addition of Br^−^ significantly enhances the fluorescence intensity of Zn(btacn)Cl_2_ by over threefold, enabling simultaneous recognition of Fe^3+^ and Br^−^. However, it should be noted that the probe’s fluorescence intensity change is not sufficiently pronounced and its detection limit is relatively low. Therefore, further structural modifications are warranted to enhance the performance of this probe.

**Figure 2.**
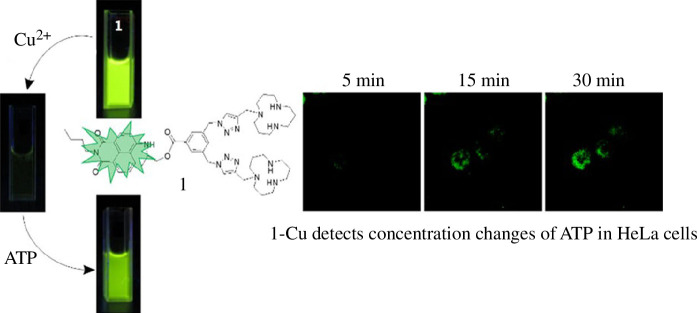
Fluorescence probe based on [12]aneN_3_ enables continuous recognition of copper ions and ATP, facilitating dynamic monitoring of intracellular ATP concentration [113]. Copyright 2016 Elsevier.

Copper is an indispensable trace element in the human body, participating in over 30 enzymes and proteins that encompass cytochrome C oxidation as well as synthesis, catalysis and metabolism of diverse enzymes within the body [41]. Furthermore, copper and zinc exhibit antagonistic properties while facilitating iron ion absorption and use [110]. Insufficient copper levels in the human body can result in elevated cholesterol, triglycerides, uric acid levels along with compromised immunity and growth retardation [111,112]. Gao *et al*. [113] synthesized a series of naphthalimide-modified fluorescent probes based on [12]aneN_3_, which exhibited remarkable water solubility. The fluorescence of the probe is reduced by 127 times upon addition of copper ions, with a lowest detection limit for copper ions reaching 1.3 × 10^−8^ M. Mechanistic investigations reveal that the coordination between two [12]aneN_3_ units and copper ions played a pivotal role in the fluorescent probe, while triazole acts as an auxiliary component. Cell experiments demonstrate that the probe possessed low cytotoxicity and excellent safety profile, enabling accurate monitoring of intracellular copper ion concentration changes. Further investigations have revealed that the [12]aneN_3_-Cu(II) complex exhibits remarkable selectivity towards ATP (figure 2), and the introduction of ATP can restore the fluorescent probe to its pre-coordination level with copper ions, indicating a competitive mechanism involving ATP in its interaction with copper ions. Through structural modifications of the probe, successful use of naphthalimide-modified [12]aneN_3_ compounds has been achieved for nucleic acid delivery, demonstrating excellent performance as a fluorescent tracer and gene transfection agent, thereby expanding the versatile applications of [12]aneN_3_ derivatives [37]. Currently, most metal ion probes are primarily used in environmental monitoring and tracking changes in metal ion concentrations within cells. However, there is limited research on monitoring alterations in metal ion concentration caused by diseases within living organisms. Consequently, there remains substantial progress to be made before clinical implementation of fluorescent probes for detecting metal ions becomes feasible.

#### 3.2.2. Bioactive small molecule probes

Bioactive small molecules, such as biological thiols, phosphate compounds, formaldehyde and carbon monoxide, play pivotal roles in the physiological activities and dynamic equilibrium of living organisms [114–116]. Biothiols are organic compounds containing sulfur that are widely distributed in biological systems and participate in various life processes including energy conversion, signal transduction and lipid metabolism [117]. The major biothiols comprise glutathione (GSH), cysteine (Cys) and homocysteine (Hcy). Despite their similar chemical structures characterized by thiol, amino and carboxyl groups presence; they exhibit distinct functions and physiological activities. GSH, with a concentration ranging from 1 to 10 mmol l^−1^, is the predominant thiol molecule in cells. It plays a crucial role in mediating the body’s oxidative stress process and safeguarding cells against oxidative metabolites and external pathogens. A decline in plasma GSH concentration serves as an important indicator for diseases such as asthma, Alzheimer’s disease and human immunodeficiency virus (HIV), while an elevation in plasma GSH level is closely associated with the onset of Parkinson’s disease [118–121].

Cys serves as a supplier of sulfur ligands in the sulfur–iron complex of organisms, participates in the synthesis of various biological enzymes and peptides, and plays a pivotal role in maintaining the tertiary and quaternary structure of proteins. In comparison to GSH, cellular Cys concentration is relatively lower, ranging from approximately 30 to 200 μM, yet it plays a crucial role in physiological activities such as cell metabolism, detoxification and protein synthesis. Abnormal levels of Cys within cells can give rise to diverse diseases including rheumatoid arthritis, oedema, growth retardation and liver damage [42,122,123]. Hcy represents an intermediate product derived from the decarboxylation metabolism of methionine during Cys production. Structurally distinct from Cys, Hcy possesses an additional methylene group. As one of the lowest-content biothiols within cells, its concentration typically ranges between 5 and 15 μM. Elevated levels often contribute to cardiovascular disease, homocysteinemia diabetes mellitus, Alzheimer’s disease and other endocrine disorders [21,124]. Wang *et al*. [125] synthesized bis[9]aneN_3_ compounds modified with 4-nitro-1,2,3-benzoxa-diazole. This complex of bis[9]aneN_3_ with mercury ions selectively stains the Golgi apparatus and exhibits selective recognition towards biothiol glutathione in human and foetal bovine serum, achieving a minimum detection limit of 0.3 μM, which is lower than that of commercial glutathione assay kits (1.0 μM). However, the probe’s sensitivity for glutathione recognition is relatively low as the addition of thiols only results in a fourfold reduction in fluorescence intensity. Therefore, this probe is not suitable for quantifying glutathione concentration in cells.

In recent years, the use of thiols' nucleophilic properties for chemical reactions with probes, resulting in a red or blue shift in the probe’s maximum emission wavelength, has emerged as a prominent research topic. Gao *et al*. [126] synthesized BODIPY modified [12]aneN_3_ through a two-step reaction using 1-acetylyl-5-chlorinated BODIPY as the starting material and characterized the product’s structure using hydrogen magnetic resonance spectroscopy (^1^H NMR), carbon magnetic resonance spectroscopy, infrared spectroscopy, and high-resolution mass spectrometry. The developed probe demonstrates simultaneous recognition ability towards Cys, Hcy and GSH. Upon addition of these three thiols, significant changes are observed in both absorption and emission spectra of the probe. Specifically, addition of GSH results in an emission colour change from green to brownish-red; addition of Hcy leads to an emission colour change from green to brownish-yellow; while addition of Cys causes an emission colour change from green to light green. Importantly, even under natural light conditions, distinct colours were observed for products formed after reacting each thiol with the probe.

The mechanistic study reveals that the sulfhydryl group in the structures of Cys and Hcy initially undergo intermolecular substitution reactions with the chlorine moiety of the probe, followed by replacement of sulfur in the molecule by the amino group in thiol through either a five-membered or six-membered ring transition state. Owing to its higher stability, the five-membered ring transition state results in a faster substitution rate between Cys and the probe compared with Hcy, enabling easy differentiation between these two thiols. By contrast, for GSH, only intermolecular substitution between its sulfhydryl group and the probe occurs without intramolecular substitution involving its amino group. This leads to the formation of BODIPY products with distinct colour and fluorescence emission properties from those resulting from amino substitution. Consequently, this probe can discriminate among all three mercaptans based on their fluorescence emission spectra and visible light characteristics. Furthermore, application of these cell imaging studies allows real-time monitoring of GSH concentration in HepG2 cells using confocal fluorescence microscopy. Developed by their research team, this reactive fluorescence probe exhibits significant changes in both fluorescence emission intensity and colour before and after reaction. Notably, it possesses excellent selectivity, high sensitivity, low detection limit, thereby demonstrating promising potential for clinical applications.

### 3.3. Diagnostic and therapeutic reagents

Iron elements play an important role in various vital biological processes, including haemoglobin synthesis, cytochrome enzyme function, peroxide metabolism, oxygen transportation in the bloodstream and DNA repair. Excessive iron accumulation can result in severe tissue damage, iron overload syndrome and carcinogenesis. Wang *et al*. [22] synthesized a series of carboxyl-modified TACN derivatives to assess their potential as iron-chelating agents against human hepatocellular carcinoma cells. In comparison to clinically used iron-depleting agent desferrioxamine and metal chelator diethylene triamine pentaacetic acid, these TACN derivatives exhibit notable anti-proliferative activity. Notably, phenylpropyl side chain-modified TACN derivatives (*p*-NO_2_-PhPr-NE_3_TA and *p*-NH_2_-PhPr-NE_3_TA) demonstrate superior anti-proliferation efficacy against human hepatocellular carcinoma cells. Regrettably, the researchers solely investigated the anti-cell proliferation activity of TACN derivatives at the cellular level with limited focus on human liver cancer cells; other cancer cell types were not explored along with tumour targeting strategies.

Obtaining disease information through the imaging function of the probe is a crucial approach for disease diagnosis. The complexes of Fe^2+^ and Fe^3+^ with macrocyclic polyamines are extensively used in biomedical imaging research [61,127,128]. Tsitovich *et al*. [43] prepared the imidazole-modified Fe(III)/TACN complex [Fe(Tim)]^3+^ and the *N*-methylimidazole-modified Fe(III)/TACN complex [Fe(Mim)]^3+^. These two low-spin Fe(III)/TACN complexes exhibit moderate paramagnetic shifts and significant ^1^H NMR responses in paraSHIFT and paraCEST applications. The magnetic moments range from 0.91 to 1.3 cm^−3^ K mol^−1^, demonstrating *S* = 1/2 state without crossover to the high-spin state within the temperature range of 0–300 K. Owing to higher ionization tendency of exchangeable NH protons in trivalent iron complexes compared with divalent transition metal ion complexes, careful selection of donor groups generating CEST is necessary. A major challenge for developing low-spin complexes as paraCEST reagents lies in significantly shifting CEST peaks while avoiding interference from magnetization transfer effects.

Boron neutron capture therapy is a precision diagnosis and treatment technology that has emerged in recent years in the field of tumour treatment. It offers outstanding clinical advantages for recurrent, invasive and locally metastatic tumours and has demonstrated significant efficacy in treating malignant brain tumours, melanoma skin cancer, osteosarcoma, breast cancer and other solid tumours [44]. The synthesis of reactive ^10^B derivatives plays a crucial role in boron neutron capture therapy, as it enables the nuclear reactions between ^10^B-containing molecules and thermal neutrons, resulting in the release of α particles and lithium atoms that effectively eradicate tumour cells. In this context, Ueda *et al*. [129] successfully designed and synthesized borate-modified [9]aneN_3_, [12]aneN_4_ and [15]aneN_5_ derivatives by linking the borate ester and polyamine through a benzene ring. Their studies demonstrate the high selectivity of single or dual-protonated [12]aneN_4_- and [15]aneN_5_-^10^B derivatives for uptake by A549 cells, where they effectively induce cell death through reaction with thermal neutrons upon interaction with zinc ions. Given the extensive use of macrocyclic polyamines as gene vectors, the safe and efficient delivery of ^10^B macrocyclic polyamines presents a novel strategy for boron neutron capture therapy.

The functionalization of chemical reagents has always been a pursuit of chemists and biologists [130]. Tang *et al*. [131] designed and synthesized four triphenylamine-modified bis-[12]aneN_3_ derivatives, which exhibited dual functionality as nucleic acid therapeutics for cancer treatment and as photosensitizers for photodynamic therapy mediated by single-electron oxygen. These compounds possess an aggregation-induced emission (AIE) property, enabling fluorescence generation in the near-infrared region (NIR) through two-photon (TP) excitation. The TP-NIR-AIE compounds demonstrate high photosensitivity under light irradiation and serve as effective cytotoxic reactive oxygen species generators, particularly singlet oxygen. Importantly, they can efficiently encapsulate DNA into nanoparticles ranging from 71 to 162 nm; longer tail hydrophobic chains of [12]aneN_3_ result in better wrapping effects on DNA and smaller particle sizes. Among the four compounds, palmitic acid-modified bis[12]aneN_3_ derivatives exhibit the highest transfection efficiency, surpassing lipofectamine 2000 by 6.7 times in HEK293T cells. Furthermore, these derivatives offer advantages such as high resolution, good penetration and enhanced safety for tumour imaging applications while also enabling production of single-electron oxygen under photoexcitation for photodynamic therapy.

**Figure 3.**
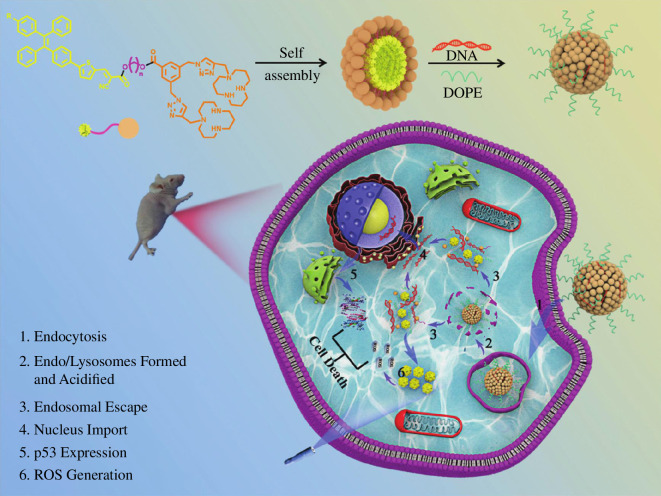
Tetraphenyl bis-[12]aneN_3_ derivatives are used in both gene therapy and photovoltaic therapy applications [132]. ROS, reactive oxygen species. Copyright 2022 Royal Society of Chemistry.

Based on this research, Liu *et al*. synthesized six types of tetraphenyl bis-[12]aneN_3_ derivatives, which were subsequently used in genetic therapy and photovoltaic therapy research (figure 3) exhibit robust AIE fluorescence emission at approximately 600 nm in aqueous solution, with Stokes shifts reaching up to 168 nm [132]. They possess the ability to encapsulate DNA into spherical particles ranging from 90 to 160 nm. Under the catalysis of acid or esterase, DNA can be released from the nanoparticles and escape its carrier. Studies have demonstrated that TTC-L-M-4 modified with lauric acid exhibits the highest transfection efficiency, surpassing lipofectamine2000 by a factor of 4.5 times. Additionally, TTC-L-M-4 demonstrates superior performance in siRNA delivery compared with lipofectamine2000, when exposed to light, TTC-L-M-4 generates reactive oxygen species for photodynamic therapy purposes. *In vitro* and *in vivo* experiments confirm that combining p53 gene therapy with photodynamic therapy significantly enhances cancer treatment efficacy. This research presents a novel method and strategy for cancer treatment.

### 3.4. Catalysts

#### 3.4.1. Water oxidation catalysts

The water oxidation reaction is extensively used in the cathode material oxidation process and represents a crucial factor for addressing energy conversion and storage challenges [62,133–135]. Owing to its high thermodynamic energy barrier, water oxidation is a complex process characterized by significant energy consumption, involving multi-electron and multi-proton transfers as well as oxygen-oxygen bond (O–O) formation. Therefore, the development of efficient catalysts for water oxidation that can enhance reaction rates is imperative for advancing energy conversion and storage technologies. In 1988, Brewer *et al*. [136] successfully synthesized Mn^2+^-cyclam and Mn^2+^-cyclen complexes, demonstrating their capability to catalytically oxidize water in acetonitrile solution containing 1.5% water, thereby establishing a pioneering example of transition metal–ligand catalysts for water oxidation.

Among various transition metal catalysts, the ruthenium complex-based water oxidation catalyst exhibits superior performance [137,138]. Younus *et al*. [139] reported the bipyridine (bpy)-modified complex, Ru-([9]aneN_3_)(bpy), and the pyridinecarboxylic acid (pic)-modified complex, Ru-[9]aneN_3_ (pic) and further investigated their photolysis in relation to their catalytic activity for water oxidation. It is observed that photolysis of Ru-([9]aneN_3_)(bpy) occurs during water oxidation, leading to the generation of electrochemically active species on its surface, while no photolysis is observed in Ru-[9]aneN_3_(pic), resulting in negligible catalytic activity. Density functional theory calculations and ^1^H NMR spectroscopy reveal that under light conditions, charge transfer between the metal Ru and ligand 2,2′-bipyridine can occur owing to a potential energy barrier being crossed in Ru-([9]aneN_3_)(bpy). Conversely, in Ru-[9]aneN_3_(pic), a higher energy barrier exists between the metal centre and pic ligand, necessitating higher energy for charge transfer [140]. This study represents, to our knowledge, the first instance of ligand-mediated water oxidation catalysis through photolysis, with a detailed investigation into its mechanism providing a novel research strategy for molecularly catalysed water oxidation.

#### 3.4.2. Olefin epoxidation catalysts

Epoxides are crucial intermediates in organic reactions and find extensive applications in resin, medicine and cosmetics [69,141,142]. The epoxidation of olefins is a common method for their synthesis, often catalysed by metal complexes, metal nanoparticles or metal oxides [70]. In recent years, there has been significant interest in developing highly efficient and selective biomimetic oxidation systems that mimic metal oxidases [143]. As non-haem enzyme mimics, iron complexes offer advantages such as high reactivity, good selectivity and mild reaction conditions for the epoxidation of olefins. The epoxidation reaction of alkenes catalysed by the non-haem mimic Fe(II)-[14]aneN_4_ complex was first reported by Nam *et al*. [144], demonstrating high yield, minimal by-products, excellent stereoselectivity and no requirement for anhydrous conditions. However, the detailed catalytic mechanism of Fe(II)-[14]aneN_4_ remains to be thoroughly investigated.

**Scheme 18.**
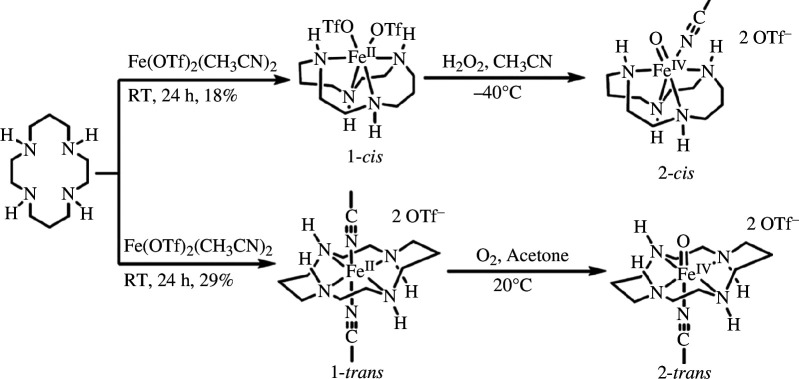
Mechanistic insights into the formation of *cis* and *trans* isomers of [14]aneN_4_ with divalent iron.

Recently, Engelmann *et al*. [145] successfully captured the intermediate of iron oxide (IV) [([14]aneN_4_)Fe(O)(CH_3_CN)]^2+^ in the epoxidation reaction catalysed by Fe(II)-[14]aneN_4_. It is characterized using mass spectrometry, X-ray absorption spectroscopy and ^1^H-NMR. The experimental results demonstrate that during the oxidation reaction of cyclohexene catalysed by Fe(II)-[14]aneN_4_, 33% hydrogen peroxide or iodosylbenzene (PhIO) preferentially oxidizes cyclohexene to epoxide rather than allyl oxidation products and dihydroxylation products. Oxoiron (IV) serves as a crucial intermediate in olefin epoxidation reactions owing to its high stereoselectivity and regional selectivity. Further investigations reveal an unreported mechanism where *trans*-oxoiron(II) complex reacts with oxygen to form *trans*-oxoiron (IV) complex through a second-order reaction in iron and a first-order reaction in oxygen (Scheme 18; [146]). In this process, *trans*-iron (III) superoxide acts as the key intermediate with higher oxygen transfer ability compared with *trans*-oxoiron (IV). It can transfer oxygen atoms to *trans*-oxoiron (II) or PPh_3_ for obtaining a *trans*-oxoiron (IV) complex. This study successfully separates and characterizes oxoiron (IV) complexes and superoxoiron (III), providing a theoretical foundation for olefin epoxidation.

#### 3.4.3. Oxygen reduction catalysts

Oxygen reduction catalysts play a pivotal role in enhancing the efficiency of fuel cells, which are widely recognized as one of the most effective solutions for global energy and environmental challenges [71,147]. Proton exchange membrane fuel cells are considered optimal power sources for electric vehicles owing to their exceptional energy power density. The anode of the battery facilitates hydrogen oxidation, while the cathode undergoes oxygen reduction reaction (ORR). Given that ORR reactions necessitate surmounting high energy barriers, it is imperative to use high-performance catalysts capable of reducing the activation energy and thereby improving battery performance. Transition metal-macrocyclic complexes such as iron-phthalocyanine, iron/cobalt-tetraphenylporphyrin and cobalt-metal organic frameworks are commonly used as oxygen reduction catalysts [63,148].

Limited research has been conducted on the ORR of macrocyclic polyamines and transition metal complexes. Recently, Vera-Estrada *et al*. [64] synthesized Rh-[14]aneN_4_ and Ru-[14]aneN_4_ complexes and investigated their electrocatalytic activity in the ORR. The findings demonstrate that both materials exhibit remarkable electrocatalytic performance irrespective of the presence of methanol. Kinetic analysis reveals that their exchange current density, indicative of charge transfer rate, surpasses that of platinum/vulcan nanoparticles. This study expands the repertoire of oxygen reduction electrocatalysts by pioneering the use of flexible macrocyclic polyamines and metal complexes as efficient catalysts for this process. However, further enhancements are still required to improve both the activity and durability of oxygen reduction catalysts, while continuous refinement in membrane electrode preparation remains crucial for achieving practical industrialization of fuel cells.

#### 3.4.4. Disproportionation catalysts (catalases)

Hydrogen peroxidase, also known as catalase (CAT), is a ubiquitous terminal oxidase found in animals, plants and microorganisms. It plays an important role as one of the key enzymes in the biological defence system established through evolutionary processes. CAT can efficiently catalyse the decomposition of hydrogen peroxide into oxygen and water, thereby mitigating oxidative stress caused by excessive reactive oxygen species within organisms [65]. CATs primarily consist of ferroporphyrin enzymes and manganese catalase. Currently, the isolation of manganese peroxide enzymes from living organisms is limited, making it a focal point for chemists and biologists to synthesize their mimics through chemical methods and investigate the catalytic disproportionation of metal ions as well as the biochemical reaction process. Manganese catalase models primarily encompass Mn-porphyrin complexes, Mn-salicylaldehyde Schiff base complexes and Mn-macrocyclic polyamine complexes. While there have been numerous investigations on Schiff base macrocyclic complexes containing phenoloxy groups, studies on Mn-macrocyclic polyamine complexes remain relatively scarce owing to the short reaction cycle of these complexes, which hinders H_2_O_2_ cycling catalysis efficiency [149].

Ren *et al*. [150] synthesized the mixed valence Mn(III/IV)-[12]aneN_4_ complex, whose structural formula was determined by X-ray single-crystal diffraction as [Mn_2_(cyclen)_2_(μ-O)_2_](ClO_4_)_3_ · 4H_2_O. Electrochemical studies reveal that this complex undergoes a single-electron redox reaction in acetonitrile solution. Recently, Freire *et al*. [151] prepared Mn-[12]aneN_4_ and Mn-pyN_3_ complexes and characterized their structures using X-ray single-crystal diffraction. The authors monitored the H_2_O_2_ decomposition process catalysed by ultraviolet-visible absorbance spectroscopy obtained structural information of the initial catalyst and intermediates, including monomers ([Mn(pyclen)Cl_2_][ClO_4_]) and dimers ([Mn_2_(pyclen)_2_(μ-O)_2_][ClO_4_]_3_). Catalytic activity studies have demonstrated that complexes containing rigid pyridine ligands, such as [Mn_2_(pyclen)_2_(μ-O)_2_][ClO_4_]_3_, exhibit higher turnover numbers and turnover frequency compared with [Mn_2_(cyclen)_2_(μ-O)_2_](ClO_4_)_3_ · 4H_2_O, indicating the crucial role of ligand structure in determining the relative activity of manganese complexes. Xu *et al*. [152] observed that introducing methyl groups on the *N* atom of the ligand enhances hydrogen peroxide decomposition reactivity, while larger substituents (*t*-butyl, cyclohexyl) hinder H_2_O_2_ disproportionation. Therefore, future catalyst research should focus on structural modification of ligands and systematic investigation of structure–activity relationships.

## 4. Conclusions and future directions

Macrocyclic polyamines serve as crucial intermediates in organic synthesis, typically obtained through a series of 3 to 5 reaction steps. The closure of macrocyclic polyamines can be achieved either by direct substitution of the amino group with halogenated hydrocarbons and sulfonates or by amidation of the amino group with the carboxyl group. Prominent synthetic approaches for macrocyclic polyamines encompass cycloaddition reactions involving dihalides and amines, cycloaddition reactions involving disulfonates and amines, as well as condensation reactions between diacids and diamines. The synthesis of macrocyclic polyamines is typically conducted in highly diluted solutions, resulting in prolonged reaction times that pose challenges for large-scale production. Additionally, the presence of various isomers complicates separation and purification processes. Although template-induced reactions have been used to overcome the limitations associated with ultra-dilute solutions, their application has mainly been limited to partial macrocyclic polyamines containing 3 or 4 nitrogen atoms. Therefore, it is crucial to explore efficient template-induced reactions and expand the range of substrates for synthesizing macrocyclic polyamines.

Macrocyclic polyamines possess a distinctive cavity structure that enables them to coordinate with heavy metal ions, transition metal ions and radioactive metal ions monitoring, medical imaging and disease treatment. The size of the macrocyclic polyamine cavity can be modulated by altering the number of nitrogen and carbon atoms, thereby influencing their coordination efficacy with diverse metal ions. By introducing alkyl chains of varying lengths on carbon atoms, the nucleic acid delivery capability of macrocyclic polyamines can be modified, subsequently impacting their transfection efficiency for DNA and RNA. However, multiple factors such as endocytosis pathways and extraction escape influence the delivery effectiveness. Henceforth, it is crucial to investigate the relationship between vector structure and transfection exploring the transfection mechanism for developing safe and efficient non-viral vectors.

The anticipated future advancements of macrocyclic polyamine compounds primarily centre around three focal points: (i) *biomedicine*: macrocyclic polyamines can serve as efficient drug delivery vehicles, targeting specific cells or tissues. A pressing challenge lies in enhancing their targeting accuracy and mitigating potential toxicities and side effects through interactions with specific biomolecules. Additionally, developing dual-function or multi-functional drug reagents incorporating diagnosis and treatment through modalities like optical and photoacoustic imaging represents a significant avenue for macrocyclic polyamines; (ii) *molecular catalysis*: macrocyclic polyamines are effective catalysts for various reactions, including addition, cyclization, REDOX and substitution reactions. Future research in this field centres on creating novel macrocyclic polyamine catalysts, elucidating their catalytic mechanisms and optimizing their performance; and (iii) *materials science*: macrocyclic polyamines, as building blocks for novel materials, can be used in the preparation of fluorescent, conductive, magnetic and other functional materials. Enhancing these materials' properties, elucidating the structure–activity relationship and guiding the design and optimization of macrocyclic polyamines are urgent research priorities. While macrocyclic polyamines have garnered significant attention in biomedicine, molecular catalysis and materials science, their commercialization remains a challenge. This review aims to provide fresh insights and perspectives, fostering further research and innovation in this exciting field.

## Data Availability

This article has no additional data.
